# Typification of Names in *Iris* (Iridaceae) Described by Victor Janka with Taxonomic Considerations

**DOI:** 10.3390/plants11131714

**Published:** 2022-06-28

**Authors:** Eugeny V. Boltenkov, Attila Mesterházy

**Affiliations:** 1 Botanical Garden-Institute, Far Eastern Branch, Russian Academy of Sciences, 690024 Vladivostok, Russia; 2 Centre for Ecological Research, Wetland Ecology Research Group, H-4024 Debrecen, Hungary; amesterhazy@gmail.com

**Keywords:** *Iris*, lectotype, neotype, nomenclature, taxonomy, typification

## Abstract

Viktor Janka von Bulcs described five names in *Iris*, i.e., *I. balkana*, *I. cretensis*, *I. lorea*, *I. mellita*, and *I. sintenisii*. These names are typified on specimens deposited at BP, JE, LD, and P, and taxonomical information is provided in the present report. Lectotypes are designated for *I. mellita* (a taxonomic synonym of *I. suaveolens*) deposited at Friedrich Schiller University Jena (JE), for *I. sintenisii* at Lund University (LD), and for *I. lorea* (a taxonomic synonym of *I. sintenisii*) at the French National Museum of Natural History (P). A neotype is designated and an image provided for the name *I. balkana* (a taxonomic synonym of *I. reichenbachii*) deposited at the Hungarian Natural History Museum (BP). The lectotype for *I. cretensis* (a taxonomic synonym of *I. unguicularis*) from JE is corrected. Images of plants of the accepted taxa are provided.

## 1. Introduction

Viktor Janka von Bulcs (1837–1900) was an Austrian botanist; his interest in botany arose during his childhood and continued to grow at high school. After meeting the Hungarian naturalist Lajos Haynald, Janka made botanical excursions with him near Cluj-Napoca, Romania [1]. In 1854, Janka joined the botanical research conducted by Philipp Johann Ferdinand Schur and Johann Mihály Fuss in Transylvania mainly near Cluj-Napoca, Sibiu, and Lugos. Additionally, he made a collecting trip to Banat with Haynald in 1856 and gathered plants in the Hungarian Great Plain in 1859 [1]. In 1870, Janka became the head of the Department of Botany, Natural History Museum of Hungary, where he served until his retirement in 1889. During this period, he explored the flora of the Balkans (including Bulgaria, Turkey, and Greece) in 1871–1872 (see, e.g., [2]) and Italy in 1874 [3,4].

Janka was a prolific author of numerous descriptions of vascular plants. At least 165 scientific names were published by Janka, and more than 30 taxa at the specific rank were published based on his own collections [5]. He collected many specimens throughout the Austro-Hungarian Empire. Most of Janka’s personal herbarium is deposited at P (French National Museum of Natural History), while a smaller part can be found at BP (Hungarian Natural History Museum) [6]; the Balkans duplicates are deposited in many herbaria [7]. In honor of his achievements in plant collections, taxonomists also used Janka’s surname for some epithets, e.g., *jankae*, *jankeana*, and *jankaeanum*, and the genus name *Jankaea* Boiss. [5].

**Figure 1:**
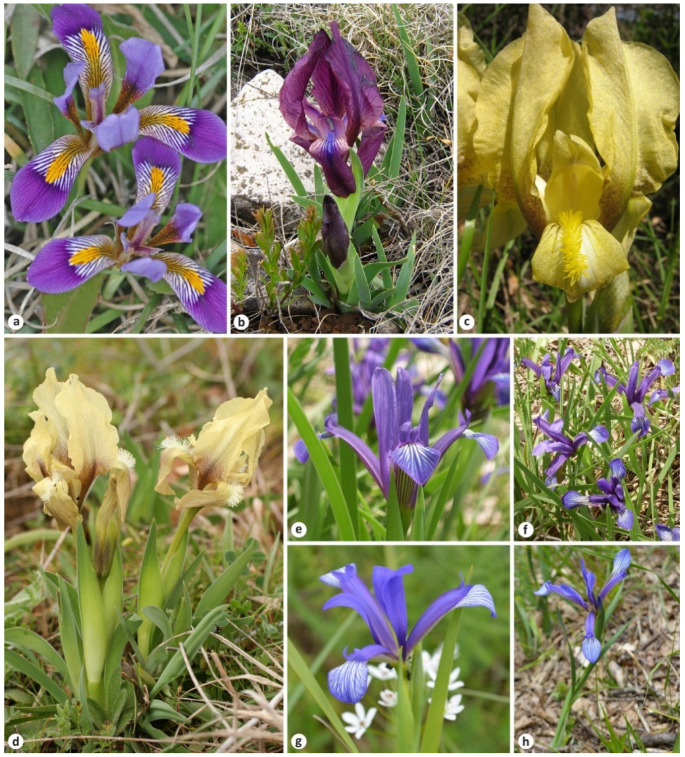
Plants associated with the names described by Victor Janka in *Iris*: (**a**) *I. unguicularis* (=*I. cretensis*) from Crete (5 km from Spili to Geraki); (**b**) the purple form of *I. reichenbachii* (=*I. balkana*) from Bulgaria (near Kralev Dol Village, Golo Bardo Mountain); (**c**) the typical yellow form of *I. reichenbachii* (south of Pernik, Golo Bardo Mountain, Bulgaria); (**d**) *I. suaveolens* (=*I. mellita*) from Bulgaria (south of Beloslav); (**e**,**f**) *I. sintenisii* from Bulgaria (southwest of Valcha Polyana, Yambol Oblast); (**g**,**h**) *I. sintenisii* (=*I. lorea*) from southern Italy (near Martina Franca, Taranto Province, Apulia); (**a**)—by A. Strid, (**b**–**f**)—by S. Stoyanov, (**g**,**h**)—by R. Labadessa.

Five species names in *Iris* L. were described by Janka in three works: *I. cretensis* Janka [8], *I. balkana* Janka and *I. mellita* Janka [9], and *I. sintenisii* Janka and *I. lorea* Janka [10] (Figure 1). Of these five names, one has been previously typified [11], and four are typified here. This paper aims to contribute to the stability of the nomenclature by typifying these *Iris* names. The present study is part of a taxonomic revision of the genus *Iris* (e.g., [12,13]).

## 2. Materials and Methods

Typification was carried out through extensive analyses of the protologues and the relevant literature, as well as by examining physical material or digital images of specimens kept at BP, JE (https://www.jacq.org/#database; accessed on 15 May 2022), K, L (https://bioportal.naturalis.nl/; accessed on 15 May 2022), LD (http://herbarium.emg.umu.se/standard_search.html; accessed on 15 May 2022), LE, P (https://science.mnhn.fr/institution/mnhn/collection/p/item/search; accessed on 15 May 2022), and PRC (https://www.jacq.org/#database; accessed on 15 May 2022) (herbarium acronyms according to Index Herbariorum [14]). Lectotypes and a neotype have been designated on the basis of relevant articles and recommendations of the *Shenzhen Code* [15] (ICN).

Janka’s *Iris* names are arranged in the chronological order of effective publication dates, including their synonyms in each entry and the information indicated in the protologue (“Protologue citation”). The accepted names of the taxa are highlighted in bold italics. Here, the conservative taxonomy of *Iris* is used [16,17,18,19,20,21,22,23]. All specimens are cited in full. All relevant material associated with the designated specimens, as well as the barcode numbers following the herbarium acronyms, were cited. Specimens were physically seen (!) unless indicated otherwise (i.e., [digital image!]). For each type designated or corrected here, direct links to specimen’s images available online are given; for one specimen unavailable online, a high-resolution image is shown. Notes on the nomenclature and taxonomy have been added for each typified name.

## 3. Results and Discussion

(1) *Iris cretensis* Janka, Oesterr. Bot. Z. 18(12): 382, 1868 ≡ *I. humilis* subsp. *cretensis* (Janka) Nyman, Consp. Fl. Eur. 4: 703, 1882 ≡ *Siphonostylis cretensis* (Janka) Wern.Schulze, Oesterr. Bot. Z. 112(3): 338, 1965 ≡ *Iris unguicularis* subsp. *cretensis* (Janka) A.P.Davis et Jury, Bot. J. Linn. Soc. 103(3): 294, 1990.—“*I. cretica* Janka”, Oesterr. Bot. Z. 18(9): 298, 1868, *nom. inval. nom. nud.* (Art. 38.1 of the ICN).—“*I. unguicularis* var. *cretensis* (Janka) Maire” in Maire et Quézel, Fl. Afrique N. 6: 151, 1959, *nom. inval.* (Art. 41.5 of the ICN).—Protologue citation: “Candia [Heraklion]”.—Lectotype (indicated by Davis and Jury [11] (p. 295), as “holotype”, corrected here): [Greece, Crete] *Iris humilis* M.B. Candia, [fl.], s.d., [*F.W. Sieber*] *s.n*. (JE00020041 [digital image!], isolectotypes BP HNHM-TRA00178014!, K000464977!, L1472079 [digital image!], LE00011043!, and PRC456611–PRC456615 [digital image!]).—https://je.jacq.org/JE00020041 (accessed on 15 May 2022).

= ***Iris unguicularis*** Poir., Voy. Barbarie 2: 86, 1789.

*Notes*—Janka described *Iris cretensis* based on three specimens sent to him by the Austrian botanist Josef Claudius Pittoni [8]. The protologue for this name includes the synonym “*I. humilis* M. a B. e Candia a Siebero divulgata”, indicating that plants were collected by Franz Wilhelm Sieber, an Austrian professional plant collector and distributor, and identified by him as *I. humilis* M.Bieb., *nom. illeg*. (Art. 53.1 of the ICN). Apparently, the plants were collected in 1817 near Heraklion (Crete, Greece), historically Candia, which was frequently mentioned by Sieber in his work [24].

Specimens of the exsiccatum “*Iris humilis* M.B. Candia”, cited in the protologue of *I. cretensis*, accompanied by a label with the printed note “*Iris humilis* M.B. Candia”, have been found at BP, JE, K, L, LE, and PRC. This exsiccatum is a part of Sieber’s botanical collection known under the title “Herbarium Florae Creticae”, issued in 1820 [25]. Davis and Jury [11] indicated that the holotype of *I. cretensis* was kept at JE as follows: “Type: Crete, Candia, 1841, *Sieber s.n*. (holotype JE!; isotype K!, P!, LE!)”. As the protologue citation does not refer to a single specimen, the term “holotype”, used by Davis and Jury [11], should be corrected to “lectotype” according to the Art. 9.10 of the ICN.

Before publishing *Iris cretensis*, two taxa were described from Crete: *I. cretica* Herb. [26] and *I. stylosa* var. *angustifolia* Boiss. et Heldr. [27]. Moreover, the original material of *I. cretica* comprises the specimen K000464977! of Sieber’s exsiccatum [28] (p. 143) also cited in the protologue of *I. cretensis*. In the following issue of *Oesterreichische botanische Zeitschrift*, after describing *I. cretensis*, Janka [29] stated that it was a synonym of *I. stylosa* Desf. or *I. unguicularis* Poir. as follows: “In der kaum gebornen *Iris cretensis* Janka fürchte ich ein Synonym von *Iris stylosa* Desf. oder *I. unguicularis* Poir. geschaffen zu haben”. In fact, *I. stylosa* and *I. unguicularis*, a priority name, were described from Algeria and are applicable to the same taxon [11,20,30].

Subsequent authors (i.e., [31,32,33]) treated *Iris cretensis* as an eastern form of *I. unguicularis* differing by the narrower leaves and by the small flowers with narrow perianth segments (Figure 1a). Dykes [16,34] noted that the broad-leaved forms of *I. unguicularis* occurred in the western and eastern part of the distribution range and suggested that dwarf plants can be a form growing on dry and poor soil. In addition, Mathew [20] saw specimens of *I. unguicularis* from Turkey and North Africa that were as small as *I. cretensis*. Hence, in *I. cretensis*, we could find neither any discontinuities in variation that are independent of environmental effects nor any geographical pattern of variation. Consequently, *I. unguicularis* is very variable in the European range and *I. cretensis* is here considered as its heterotypic synonym [16,17,18,19,20,35,36,37].

**Figure 2:**
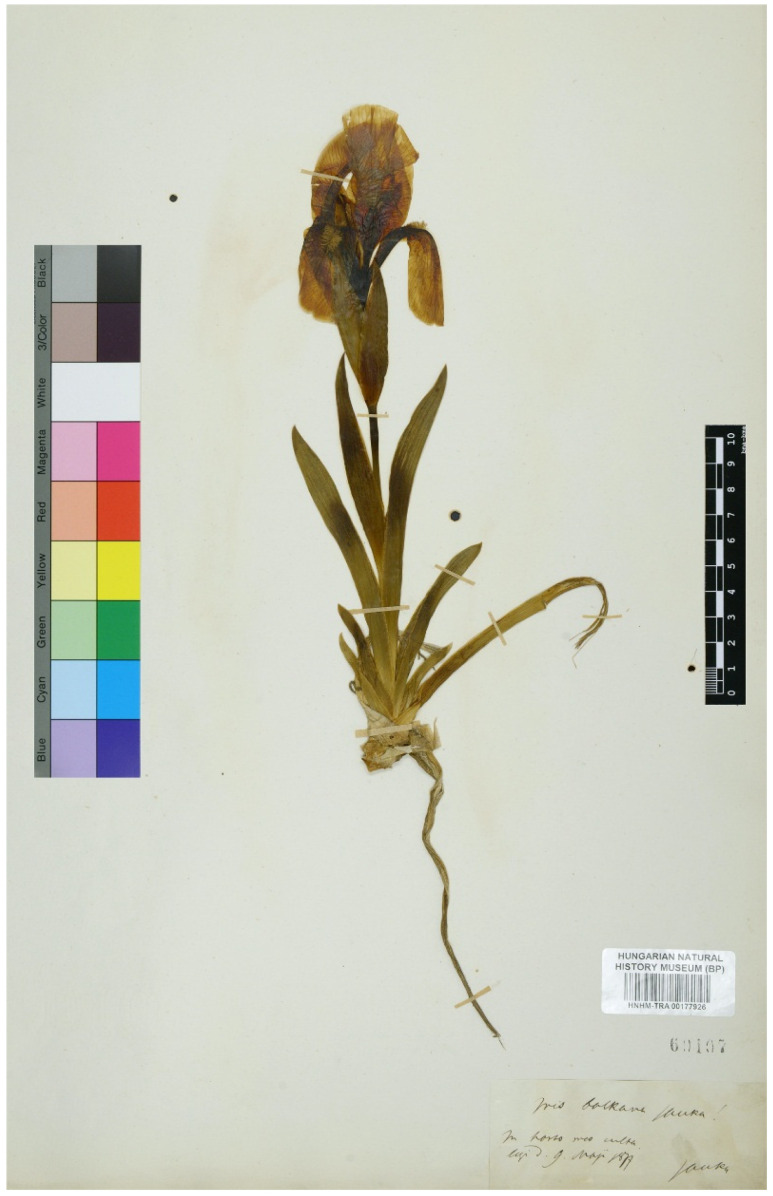
Neotype of *Iris balkana* (BP HNHM-TRA00177926), by permission of the Curator.

(2) *Iris balkana* Janka, Mat. Term. Közlem. 12: 173, 1876 ≡ *I. chamaeiris* var. *balkana* (Janka) Baker, Gard. Chron., n.s., 6: 648, 1876 ≡ *I. chamaeiris* subsp. *balkana* (Janka) K.Richt., Pl. Eur. 1: 254, 1890 ≡ *I. reichenbachii* var. *balkana* (Janka) Acht., Izv. Bot. Inst. (Sofia) 7: 34, 1960.—Protologue citation: [origin not specified].—Neotype (designated here): [Specimen from a cultivated plant] *Iris balkana* Janka! In horto meo culta, [fl.], legi d. 9 May 1877, *Janka s.n*. (BP HNHM-TRA00177926!).—Figure 2.

= ***Iris reichenbachii*** Heuff., Oesterr. Bot. Z. 8(1): 28, 1858.

*Notes*—*Iris balkana* was described by Janka without indicating any locality of collection [9]. In the section for the southeastern part of Hungary (“Adatok Magyarhon délkeleti vírányálioz”), Janka compared *I. pumila* L. with similar species and, for this reason, included here *I. balkana* and *I. mellita* (see below). Subsequently [10], Janka provided a more detailed description of *I. balkana*, also providing a colored illustration and asserted “Habitat in locis saxosis graminosis regionis mediae m. Balkan Thraciae borealis supra pag. Kalofer, … detexi d. 27. Maji 1871. Plantam vivam attuli, in horto meo nune laetissime vigentem”. According to this, *I. balkana* was discovered by Janka near Kalofer, located in the historical region of Northern Thrace, central Bulgaria, and then introduced into cultivation.

In the present study, we searched the specimens of *Iris balkana* with Janka’s annotations and have found two specimens, BP HNHM-TRA00177926! and BP HNHM-TRA00177929!. These are dated 1877, i.e., after publication of *I. balkana*, and are therefore not the original material for the name. During World War II, some parts, including several types of BP collection, were moved to Alsópetény Village (Hungary), and most of them were destroyed there [38]. Most probably, the original material of *I. balkana* was included in this collection. Unfortunately, the original material, on which this name was based, has not been found in other examined herbaria. Therefore, a neotype may be selected according to the Art. 9.13 of the ICN. The specimen BP HNHM-TRA00177926! (Figure 2) is designated here as a neotype for *I. balkana*, since it is the most informative.

Janka [10] noted that *Iris balkana* is rather related to *I. reichenbachii* Heuff. However, Baker [39,40] assumed *I. balkana* to be a variety of *I. chamaeiris*. On the contrary, Armitage [41] reasonably pointed out that the foliage in *I. balkana* only appears in spring, and its distribution range is Eastern Europe. In fact, *I. chamaeiris* is a synonym of *I. lutescens* Lam., an evergreen species that is native to southern Europe, with its distribution range extending from Portugal to northwestern Italy [42]. Subsequently, *I. balkana* was regarded as a purple form or variety of *I. reichenbachii* [17,33,43,44] or as a synonym of the latter [19,20,45,46]. *Iris reichenbachii* is a Balkan endemic mainly common in the montane parts of Bulgaria, Montenegro, Serbia, North Macedonia, and northeast Greece. According to Mitra [47], the karyotypes of the 24-chromosome *I. reichenbachii* and *I. balkana* are similar. In contrast to yellow-flowered *I. reichenbachii*, *I. balkana* is purple-flowered, with blue beard hairs (Figure 1b,c). Some populations in Bulgaria consist of yellow-flowered or purple-flowered forms only (e.g., Golo Bardo Mountain), while in some others (e.g., the Rhodope Mountains), purple and yellow forms occur together (S. Stoyanov, pers. comm.).

(3) *Iris mellita* Janka, Mat. Term. Közlem. 12: 172, 1876 ≡ *I. rubromarginata* subsp. *mellita* (Janka) K.Richt., Pl. Eur. 1: 254, 1890 ≡ *I. rubromarginata* var. *mellita* (Janka) Hayek, Repert. Spec. Nov. Regni Veg. Beih. 30(3): 120, 1932.—“*I. pumila* subsp. *mellita* (Janka) Beldie”, Fl. Român. 2: 273, 1979, *nom. inval.* (Art. 41.5 of the ICN).—Protologue citation: [origin not specified].—Lectotype (designated here): [Bulgaria] *Iris mellita* Janka “Adatok” (1874) … In declivisis merid. collis Tschierdem-Tepe prope Philippopolim argillosis ad vineorum marginis, [fr.], legi d. 1 July 1871, *Janka s.n.* (JE00022413 [digital image!]).—https://je.jacq.org/JE00022413 (accessed on 15 May 2022).

= ***Iris suaveolens*** Boiss. et Reut., in Boiss., Diagn. Pl. Orient., ser. 1, 2(13): 15, 1854.

*Notes*—Originally, *Iris mellita* was described by Janka without indicating the collection locality [9]. In his following work [10], Janka provided the necessary clarifications as follows: “Habitat in herbidis aridis infra cacumen collis «Tschiendem-Tepe» prope Philippopolin Thraciae, ubi fructibus maturis d. 1. Julii 1871 … E seminibus coleo …”. According to this, *I. mellita* was first collected by Janka near Philippopolis, the former name for the modern city of Plovdiv located in the Northern Thrace, central southern Bulgaria, and then introduced into cultivation. Hence, we consider the name *I. mellita* to be based on specimens in fruiting, collected in Plovdiv Province, Bulgaria, in 1871, and plants raised from seeds by him.

The specimen JE00022413 is designated here as the lectotype of *Iris mellita* because it matches the information from Janka’s work [10] and is certainly the original material for the name. The original label handwritten by Janka and identified by him as “*Iris mellita* Janka” accompanies this specimen. The following information from the label “Adatok 1874” corresponds to Janka’s work [9].

The taxonomy of *Iris mellita* has been a source of confusion. In particular, Janka [10] associated his *I. mellita* with *I. rubromarginata* Baker described a year earlier, and, as a result, *I. mellita* was attributed to *I. rubromarginata* at the rank of subspecies [48] or variety [49]. Subsequently, *I. mellita* was recognized as a distinct species, including *I. rubromarginata* and *I. straussii* Micheli [16,43,50]. On the contrary, Peckham [45] treated *I. mellita* as a synonym of *I. straussii*. However, it has been established that the last-named *I. rubromarginata*, *I. mellita*, and *I. straussii* are taxonomic synonyms of *I. suaveolens* Boiss. et Reut. [20,21,23,46,51]. It is a dwarf relative of *I. reichenbachii*, differing mainly by the more rigid keeled bracts, longer perianth tube, and shorter stem (Figure 1d), distributed in the eastern Balkans from Macedonia to western and northern Turkey.

(4) ***Iris sintenisii*** Janka, Természetrajzi Füz. 1: 244, 1877 ≡ *I. graminea* subsp. *sintenisii* (Janka) K.Richt., Pl. Eur. 1: 256, 1890 ≡ *Chamaeiris sintenisii* (Janka) M.B.Crespo, Flora Montiber. 49: 66, 2011.—“*Xyridion sintenisii* (Janka) Rodion.”, Bot. Zhurn. (Moscow and Leningrad) 90(1): 58, 2005, *nom. inval.* (Art. 41.5 of the ICN).—Protologue citation: “Habitat in m. Balkan Thraciae borealis supra Slivno (Fridvadszky); in Bulgariae orientalis districtu Dobrudscha, ubi legit amic. P. Sintenis.—In cretaceis elatioribus versus cacumen m. «Tschatalkaje» prope Slivno ego ipse d. 4. Augusti 1872”.—Lectotype (designated here): [Romania] *Iris sintenisii* Janka, Babadagh: Waldränder zwischen Baschbunar u. Teke, [fl.], 9 Juni 1874, *Sintenis 799*, [stamp] Herb. P. Sintenis (LD1213150 [digital image!], isolectotype BP HNHM-TRA00155284!, Herb. Haynald).—http://www.botmus.lu.se/Lund/Images/1213150.jpg (accessed on 15 May 2022).

*Notes*—*Iris sintenisii* was described based on three gatherings, of which two were collected near Sliven, Bulgaria, by Imre Frivaldszky and by Janka, and the third one was collected by the German plant collector Paul Ernst Emil Sintenis, whom the specific epithet *sintenisii* honors, in the Dobrudja, a historical region in the Balkans [10]. We found two specimen of *I. sintenisii*, collected by Sintenis from Northern Dobrudja between Fântâna Mare and Mina Altan-Tepe, Romania, in his first expedition [52]. The specimen from Sintenis’ personal herbarium in the Botanical Museum at Lund University (LD1213150), accompanied by a printed label with the note “Gebr. Sintenis Reise in der Dobrudscha” and handwritings made by Sintenis (also see [53]), is designated here as lectotype because it matches the protologue and is the complete one.

After describing, *Iris sintenisii* has become the accepted name for the plant distributed in Albania, Bulgaria, northern Greece, southern Italy, Moldova, North Macedonia, Romania, southeastern Serbia (very rare), northwestern Turkey, and western Ukraine (Chernivtsi Oblast) [16,19,20,23,54,55,56,57]. This plant usually occurs in dry meadows, on scrubland, and at forests’ edges (Figure 1e,f). *Iris sintenisii* is close to *I. graminea* L., from which it may be readily distinguished by the following features: smaller size; rosette leaves firmer, acuminate, narrow, 0.3–0.6 cm wide (vs. 0.5–1.5 cm wide); stem terete (vs. flattened); outer bract slightly shorter than inner bract (vs. outer bract rather longer than inner bract and leaf-like); perianth tube up to 0.9 cm long (vs. 0.5 cm long); fruit with coupled ribs in 3 pairs and slender beak to 2 cm long (vs. with 6 conspicuous, equidistant ribs, and apex shortly beaked, up to 0.5 cm long).

(5) *Iris lorea* Janka, Természetrajzi Füz. 1: 245, 1877 ≡ *I. foetidissima* subsp. *lorea* (Janka) K.Richt., Pl. Eur. 1: 258, 1890 ≡ *Xiphion gramineum* subsp. *loreum* (Janka) Arcang., Comp. Fl. Ital., ed. 2: 157, 1894 ≡ *Iris foetidissima* var. *lorea* (Janka) Fiori, Fl. Italia [Fiori, Béguinot and Paoletti] 4(App.): 51, 1907 ≡ *Chamaeiris sintenisii* subsp. *lorea* (Janka) M.B.Crespo, Flora Montiber. 49: 66, 2011 ≡ *Ch. lorea* (Janka) Peruzzi, F.Conti et Bartolucci, Inform. Bot. Ital. 46(2): 276, 2014.—Protologue citation: “Habitat in paludosis maritimis districtus “Terra d’Otranto” Italiae meridionales, ubi aestata a. 1875 legerunt dd.*Porta et Rigo*”.—Lectotype (designated here): [Italy] *Iris foetidissima* auctor. Italic, caule tereti, Italia austral., Apulia in paludosis pr. Cerignola, [fl.], 6 June 1875, *Porta et Rigo s.n.* Exs. no. 382 (P02167368 [digital image!], isolectotypes BP HNHM-TRA00178065!, K!, P02167280, and P02167287 [digital images!]).—http://coldb.mnhn.fr/catalognumber/mnhn/p/p02167368 (accessed on 15 May 2022).

= ***Iris sintenisii*** Janka, Természetrajzi Füz. 1: 244, 1877.

*Notes*—According to the protologue [10], the name *Iris lorea* was based on two specimens given to Janka from Rupert Huter and originally identified as *I. foetidissima* L. as follows: “Specimina duo mihi transmisit cl. Huter sub nomine falso: ‘*Iris foetidissima* autorum florae Italiae’”. The plants were gathered in the district Terra d’Otranto during the second joint journey of Pietro Porta and Gregorio Rigo in 1875. The area of Terra d’Otranto is a geographical region of Apulia, Italy, largely corresponding to the Salento Peninsula.

Clementi [58] noted that the type of *Iris lorea* had not been designated. At BP, K, and P, we have found five duplicates that match the information from the protologue and are, hence, the original material for the name. The specimen P02167368 is designated here as lectotype because it is the most informative one. Huter identified and distributed the herbarium specimens collected by Porta and Rigo; however, the date and the collection number during the journey of Porta and Rigo to Apulia in 1875, as well as the collector’s information, are often missing [59]. The specimens accompanied by the printed labels, annotated as “№ 382. Porta et Rigo ex itinere II italico”, were identified by Huter as follows: “*foetidissima* auctor. Italic”. Therefore, we believe that these specimens refer to the exsiccatum issued by Huter. As it follows from the contents of the labels, the plants of this exsiccatum were collected near Cerignola in the Foggia Province, Italy, and the flowering stems were terete.

Janka [10] noted that *Iris lorea*, while being comparable with *I. sintenisii* by the elongated, terete stem, and elongated perianth tube, however, differed from the latter by its pale green and very long leaves, much longer than the stem, and by the herbaceous, green bracts. The etymology of the specific epithet *lorea* (from the Latin *loreus*) is from the lorate, or strap-shaped, rosette leaves. The lorate leaves are also characteristic of *I. sintenisii* (see above). We are inclined to suggest that the morphological differences between *I. lorea* and typical *I. sintenisii* are few. As regards the habitat of *I. lorea*, it was found in a swampy coastal area, which is not a common condition of the xerophilous *I. sintenisii*. However, smaller plants than those of *I. lorea*, i.e., typical of *I. sintenisii*, occur in dry places in Apulia (Figure 1g,h; see also the image [60]).

Janka [10] simultaneously published *Iris sintenisii* and *I. lorea*; however, Dykes [16] established priority of *I. sintenisii* (see Art. 11.5, Note 3 of the ICN). Both of these names have been referred to the same taxon, *Xiphion collinum* (N.Terracc.) [61] or *Iris graminea* var. *collina* (N.Terracc.) Fiori [62]. After the combinations based on *I. lorea* were proposed 1907 (see above), this taxon was not mentioned in the literature for a long time, except for a mention by Prodan [63]. Since 2007, it has been actually reinstated for the Italian flora and cited as *I. lorea* [64,65] or *Chamaeiris lorea* [60,66,67,68], often including *I. sintenisii* as a synonym. However, although the plants from Italy are claimed to be distinct, *I. lorea* closely resembles *I. sintenisii* and should be regarded as a synonym of it [19,20,22,33,45], which we completely support. In southern Italy, *I. sintenisii* is distributed in the regions of Abruzzo, Apulia, Basilicata, Calabria, Campania, and Molise.

## Data Availability

Not applicable.
